# Analysis of microbial community structure and volatile compounds in pit mud used for manufacturing Taorong-type Baijiu based on high-throughput sequencing

**DOI:** 10.1038/s41598-022-10412-8

**Published:** 2022-05-05

**Authors:** Yanbo Liu, Mengxiao Sun, Pei Hou, Wenya Wang, Xiangkun Shen, Lixin Zhang, Suna Han, Chunmei Pan

**Affiliations:** 1 College of Food and Biological Engineering (Liquor College), Henan University of Animal Husbandry and Economy, Zhengzhou, 450046 China; 2 Postdoctoral Programme, Henan Yangshao Distillery Co., Ltd., Mianchi, 472400 China; 3 School of Life Sciences, Henan University, Kaifeng, 475004 China; 4 Henan Liquor Style Engineering Technology Research Center, Henan University of Animal Husbandry and Economy, Zhengzhou, 450046 China; 5 Zhengzhou Key Laboratory of Liquor Brewing Microbial Technology, Henan University of Animal Husbandry and Economy, Zhengzhou, 450046 China; 6 School of Food and Bio-Engineering, Zhengzhou University of Light Industry, Zhengzhou, 450000 China; 7 Henan Food Industry Science Research Institute Co., Ltd., Zhengzhou, 450003 China

**Keywords:** Biotechnology, Microbiology

## Abstract

In this study, the pit mud used in manufacturing Taorong-type Baijiu was collected from the upper, middle, lower and bottom layers of pits at Henan Yangshao Liquor Co., LTD. High-throughput sequencing (HTS) technology was used to analyze the microbial community structure of the pit mud. In addition, the volatile compounds in the pit mud were subjected to preliminary qualitative analysis through headspace-solid phase microextraction and gas chromatography–mass spectrometry (GC–MS). The HTS results demonstrated that there were 5, 3, 5 and 5 dominant bacterial phyla (including 11, 11, 9 and 8 dominant bacterial genera) and 3, 3, 3 and 3 dominant fungal phyla (including 4, 7, 7 and 5 dominant fungal genera) in the pit mud from the F-S (upper), G-Z (middle), H-X (lower) and I-D (bottom) layers, respectively. In the qualitative analysis of the volatile compounds, a total of 77types of volatile compounds were detected in the pit mud, including 46, 45, 39 and 49 types in the pit mud from layers F-S, G-Z, H-X and I-D, respectively. Esters and acids were the two main components of the pit mud. The correlation between the microorganisms present and the main volatile compounds in the pit mud was analyzed. *Lentimicrobium*, *Syner-01* and *Blvii28_wastewater-sludge groups* were found for the first time in pit mud used for manufacturing Taorong-type Baijiu. The findings of this study could provide a theoretical foundation for improving the quality of pit mud and the flavor of Taorong-type Baijiu.

## Introduction

Baijiu has a long history in China, and it is one of China's national cultural hallmarks^1^. As an innovative flavor type in this industry in China, Taorong-type Baijiu is uniquely characterized by a "yellowish and transparent color, delicate, mellow and harmonious flavor and lasting fragrance”^2^. There is an old saying, namely, "pits throughout years and distillers' grains throughout centuries". Taorong-type Baijiu is fermented in a solid anaerobic environment^3^. Pit mud is the basis for the fermentation of traditional solid-state Baijiu^4^. As one of the crucial influencing factors, pit mud determines to a great extent the quality and flavor of Baijiu produced^5^ and plays a vital role in the brewing process of Taorong-type Baijiu^6^. Pit mud also provides a suitable habitat for fermentation microorganisms^5^ that grow and reproduce in pit mud^7^. The microbial communities present in pit mud constitute a complex ecosystem^8^. The substances that give Taorong-type Baijiu its flavor are mainly generated in pit mud. There are different living environments for the microorganisms in different spatial positions of the pits^9^. Various microbial communities contribute to generating specific flavor compounds that in turn determine the flavor and quality of Baijiu^10^. In addition, the pottery cellar of Taorong liquor is different from that of other fragrant liquors. It is about 3 m deep. Compared with other cellars, in terms of thermal insulation performance, the pottery cellar has thicker walls and better heat absorption and heat dissipation, so it is less affected by external temperature and climate changes; in terms of incense-producing function, the microporous channels in the pottery slices The special structure of He Tao mud is extremely beneficial to the enrichment, respiration and reproduction of brewing microorganisms and aroma-producing functional bacteria.

The microbial diversity of pit mud has a significant influence on the flavor of Baijiu^11^. Bacteria and fungi are important functional flora that produce abundant enzymes and flavor substances contained in Baijiu, thus bestowing it with unique flavor^12^. Bacterial metabolism in pit mud generates important organic acids (such as butyric acid and caproic acid) in Taorong-type Baijiu that increase its flavor and reduce its stimulation. Fungi such as *Saccharomyces cerevisiae* can be involved in alcoholic fermentation, and *Aspergillus* can be involved in the generation of glucoamylase. The composition and quantity of microorganisms in pit mud are two of the factors that affect the flavor of Taorong-type Baijiu^13^. Pit mud in different spatial positions could affect the reproduction and metabolism of microorganisms and the flavor composition of Taorong-type Baijiu^14^. Therefore, it is necessary to explore the microbial communities present in pit mud.

Traditionally, isolation and culture methods are used in the study of microorganisms^15^. However, because the species and quantities of isolated microorganisms are limited, many microbial species are often missed, and key functional microorganisms may even be omitted^16^. Because the closed environment of pit mud is strictly anaerobic in essence, many microorganisms cannot be cultivated or are difficult to cultivate. Nonculture methods that objectively reflect the microbial composition of samples can be employed to detect more microorganisms than can be detected using culture methods^17^. Among nonculture methods, high-throughput sequencing (HTS) technology is the preferred method for the analysis of pit mud^18,19^. HTS methods have been extensively used in an attempt to obtain a more comprehensive analysis of microbial diversity^20^. HTS, also known as next-generation sequencing (NGS), features high throughput, high sensitivity, high resolution, low cost and simple operation; thus, it can be employed to generate large amounts of data in a short time^21^ and to obtain comprehensive information on microbial diversity and microbial community composition. Headspace solid-phase microextraction (HS-SPME) combined with gas chromatography–mass spectrometry (GC–MS) is an advanced technology^22^ that can be very effective in the analysis of volatile substances^23^. Solid-phase microextraction (SPME) is a rapid analysis technology that integrates the pretreatment, adsorption and extraction of samples. Because various types of adsorbents with different polarities are available, it is possible to separate trace compounds present in a variety of substrates, and this method has been used in a number of fields. In addition, SPME is suitable for the detection and analysis of trace components, process monitoring and other purposes in the production of Baijiu and in related processes^24^. Gas chromatography–mass spectrometry (GC–MS) has been regarded as the gold standard for the analysis of many compounds^25^. Furthermore, it is a common and mature technique that is used in the analysis of volatile and semivolatile aromatic components worldwide. GC–MS has advantages such as high sensitivity, high separation efficiency, high selectivity and rapid analysis. It offers excellent performance in separation, detection and data processing and hence can be used to perform accurate qualitative analyses^2^.

There are currently no reports on the microbial community structure in pit mud used to manufacture Taorong-type Baijiu or on the volatile compounds present in this type of mud. In this study, HTS technology was used to analyze the microbial community structure of the upper, middle, lower and bottom layers of pits used in manufacturing Taorong-type Baijiu. In addition, the volatile compounds present in pit mud from different layers were investigated by HS-SPME and GC–MS. The correlation between the microbial community structure and the presence of specific volatile compounds was explored, and the results provide a resource for establishing a microbial information database for Taorong-type Baijiu. In addition to providing theoretical support for the development of methods to improve the quality of pit mud, cultivate artificial pit mud, and improve the flavor and quality of Baijiu, the results of this study contribute to a thorough understanding of the contribution of microbes present in pit mud to the flavor that develops during the brewing of Baijiu.

## Materials and methods

### Materials

The pit mud samples used in this experiment were obtained from 30-year-old pit mud at Henan Yangshao Liquor Co., LTD. Three pits were randomly selected, and 10 g of pit mud was collected from the centers of each of the four pit walls of the upper layer (F-S, 50 cm from the pit mouth), the middle layer (G-Z, the pit center) and the lower layer (H-X, 50 cm from the pit bottom) as well as from the center of the bottom layer (I-D) of each pit. The samples obtained from each wall of each layer of the individual pits were mixed evenly. The collected samples were designated F-S-1, F-S-2, F-S-3, G-Z-1, G-Z-2, G-Z-3, H-X-1, H-X-2, H-X-3, I-D-1, I-D-2 and I-D-3. They were stored in a refrigerator at – 20 °C^26^.

### Reagents and instruments

Reagents: D3141 HiPure Soil DNA Kits (Soil DNA Extraction Kit) were purchased from Guangzhou Magen Biotechnology Co., Ltd. PCR-related reagents were purchased from TOYOBO (SHANGHAI) BIOTECH CO., LTD. AMPure XP magnetic beads were purchased from Beckman Coulter, USA. Anhydrous ethanol was purchased from Guangzhou Chemical Reagent Factory (GCRF). Agarose (BiowestAgarose) was purchased from Beijing Mengyimei Business Center. Goldview (Goldview I) was purchased from Beijing Mengyimei Business Center. NaCl was purchased from Tianjin Kemiou Chemical Reagent Co., Ltd.

Instruments: A centrifuge (Eppendorf 5427R) was purchased from Eppendorf AG, Germany. A pipette (Eppendorf) was purchased from Eppendorf AG, Germany. An ultrapure water instrument (Mingche TM-D) was purchased from RephiLe Bioscience Ltd., Shanghai. A refrigerator (− 80 °C) (DW-HL528S) was purchased from Zhongke Meiling Cryogenics Co., Ltd. A vortex oscillator (mix-28+) was purchased from Guangzhou Wego Instrument Co., Ltd. A NanoDrop spectrophotometer (NanoDrop 2000) was purchased from Thermo Fisher Scientific, USA. An agarose gel electrophoresis apparatus (DYY-6C) was purchased from Beijing Scientific Biotechnology Co., Ltd. A gel imaging system (Tanon-2500) was purchased from Tanon (Shanghai); a thermal cycler (ETC811) was purchased from EASTWIN Scientific Instruments Inc. Qubit 3.0 was purchased from Thermo Fisher Scientific. A gas chromatography–mass spectrometry system (GCMS-QP2010 Ultra) was purchased from Shimadzu, Japan. A solid-phase microextraction device was purchased from Merck & Co., Inc., USA (Supplementary Figs. 8, 9, 10, 11).

### Experimental methods

#### Extraction of DNA from samples

According to the instruction manual provided with the HiPure Soil DNA Kit (Soil DNA Extraction Kit) from Guangzhou Magen Biotechnology Co., Ltd., the genomic DNA of bacteria was extracted; the integrity of the extracted DNA was measured by 1% agarose gel electrophoresis.

#### PCR amplification

The primers used to amplify bacterial DNA were 341F (5′-CCTACGGGNGGCWGCAG-3′) and 806R (5′-GGACTACHVGGGTATCTAAT-3′); those used to amplify fungal DNA were ITS3_KYO2F (5′-GATGAAGAACGYAGYRAA-3′) and ITS4R (5′-TCCTCCGCTTATTGATATGC-3′).

#### First round of amplification

The system used in the first round of amplification included 10 × Buffer KOD, 5 μL; 2 mM dNTPs, 5 µL; 25 mM MgSO_4_, 3 µL; Primer F (10 μM), 1.5 µL; Primer R (10 μM), 1.5 µL; KOD enzyme, 1 µL; template, 4 µL (100 ng); H_2_O, to 50 µL. The procedure used in the first round of amplification was 94 °C/2 min, 98 °C/10 s, 62–66 °C/30 s, and 68 °C/30 s (30 cycles) followed by 68 °C/5 min. The PCR product was purified using AMPure XP Beads and quantified by Qubit 3.0. After the first round of amplification, a second round of amplification was performed.

#### Second round of amplification

The system used in the second round of amplification included 10 × Buffer KOD, 5 μL; 2 mM dNTPs, 5 µL; 25 mM MgSO_4_, 1 µL; Index Primer (10 μM), 1 µL; Universal PCR Primer (10 μM), 1 µL; KOD enzyme, 1 µL; template, 4 µL (100 ng); H_2_O, to 50 µL. The procedure used in the second round of amplification was 94 °C/2 min, 98 °C/10 s, 65 °C/30 s, and 68 °C/30 s (12 cycles) followed by 68 °C/5 min. The PCR products obtained in the second amplification were detected by agarose gel electrophoresis.

#### Library quantification and sequencing

AMPure XP Beads were used to purify the products of the second round of amplification, and an ABI StepOnePlus Real-Time PCR System (Life Technologies) was used to conduct the quantification. Relying on Guangzhou Gene Denovo Biotechnology Co., Ltd., the products were sequenced on a Novaseq 6000 using PE250 mode pooling.

#### Pretreatment of pit mud samples

One gram of pit mud was placed in a headspace bottle, and 2 g NaCl and 5 mL distilled water were added. The bottle was then tightly stoppered, and the mixture was shaken thoroughly.

#### Conditions for headspace solid-phase microextraction (HS-SPME)

A 1 g pit mud sample was weighed and placed in a headspace bottle, which was then preheated in a water bath at 50 °C for 10 min. A solid-phase CAR/PDMS (75 μm CAR/PDMS, carbon molecular sieve/polydimethylsilane) extraction fiber head was inserted into the silica gel stopper of the headspace bottle and inserted into the sample for headspace adsorption for 30 min.

#### Conditions of gas chromatography–mass spectrometry (GC–MS)

The conditions used in gas chromatography were as follows: HP-FFAP chromatographic column (30 m × 0.32 mm × 0.25 μm); no shunt; flow rate 1.21 mL/min; temperature at sample injection port 250 °C; heating at 40 °C for 3 min, 5 °C/min for 60 °C without holding, and 8 °C/min to 230 °C for 7 min.

The conditions used in mass spectrometry were as follows: interface temperature 220 °C; ionization mode electron ionization (EI) source; electron energy 70 eV; ion source temperature 200 °C.

## Results

### Basic sequencing data and alpha diversity analysis

As shown in Table 1, effective sequencing was achieved through quality control of original sequences and removal of chimeras. The average numbers of remaining effective sequences obtained from the pit mud samples from F-S, G-Z, H-X and I-D are 116,693, 122,327, 115,977 and 12,1991, respectively. The coverage rate is greater than 0.99, indicating that the sequencing depth is sufficient; the sequences in the samples are basically completely detected, and the results are true and reliable and can be used in the subsequent analysis.

**Table 1:** Basic sequencing data obtained from pit mud from F-S, G-Z, H-X and I-D.

Sample	16S				ITS			
	Effective sequence	Shannon index	Chao 1 index	Coverage rate	Effective sequence	Shannon index	Chao 1 index	Coverage rate
F-S-1	112,576	5.2948	826	0.9976	128,083	2.4599	86	0.9999
F-S-2	122,754	5.3320	842	0.9978	120,498	2.4471	91	0.9999
F-S-3	114,750	5.3160	812	0.9979	120,712	2.6153	92	0.9998
G-Z-1	127,751	5.4721	785	0.9983	117,690	3.1094	89	0.9998
G-Z-2	119,701	5.4386	773	0.9982	117,107	2.5827	87	0.9999
G-Z-3	119,528	5.3970	762	0.9981	117,972	2.2831	61	0.9999
H-X-1	117,698	5.2033	815	0.9978	126,473	3.6627	101	0.9998
H-X-2	116,475	5.2284	754	0.9978	123,248	3.5273	93	0.9999
H-X-3	113,759	5.1491	768	0.9976	121,489	3.4006	98	0.9999
I-D-1	125,031	4.3747	815	0.9979	126,599	3.1670	93	0.9999
I-D-2	120,550	4.5248	800	0.9978	117,278	3.2523	102	0.9999
I-D-3	120,392	4.4931	804	0.9979	126,217	3.1045	100	0.9999

F-S, upper layer; G-Z, middle layer; H-X, lower layer; I-D, bottom layer.

In alpha diversity analysis of pit mud samples, the Chao 1 index is mainly related to the abundance of samples; the larger the Chao 1 index is, the higher the abundance is. The Shannon index is mainly related to the diversity of samples; it reflects not only the abundance of species but also the evenness of species. The larger the Shannon index is, the higher the diversity is. As seen from Table 1, in terms of the 16S rDNA sequence, the samples can be ranked as F-S > I-D > H-X > G-Z with respect to their Chao 1 index values and as G-Z > F-S > H-X > I-D with respect to their Shannon index values. In terms of the ITS sequence, the samples rank as I-D > H-X > F-S > G-Z in Chao 1 index values and as H-X > I-D > G-Z > F-S in Shannon index values.

### Venn diagram analysis

**Figure 1:**
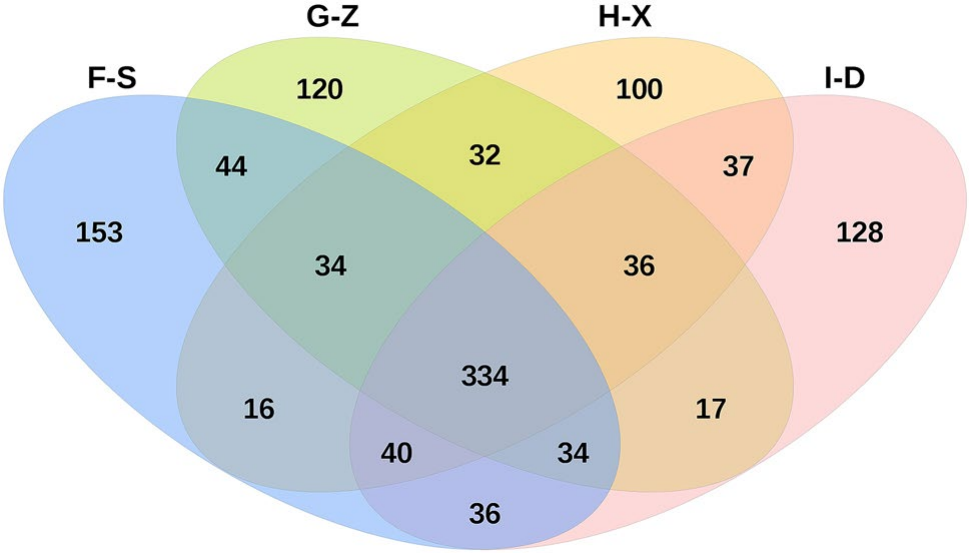
Venn diagram showing the numbers of operational taxonomic units found in pit mud from F-S, G-Z, H-X and I-D.

Figure 1 presents a Venn diagram based on OTUs (operational taxonomic units). The overlapping parts of the differently colored areas in the Venn diagram represent the numbers of common species present in the samples, while the nonoverlapping parts represent the numbers of unique species in the samples. The diagram clearly shows the similarities and differences among the samples. As shown in Fig. 3, the numbers of OTUs found in the pit mud from F-S, G-Z, H-X and I-D were 691, 651, 629 and 662, respectively. The total number of common OTUs shared by the four pit mud samples is 334, indicating that 334 bacterial species exist simultaneously in the pit mud from F-S, G-Z, H-X and I-D. The numbers of unique OTUs in the pit mud from F-S, G-Z, H-X and I-D are 153, 120, 100 and 128, respectively.

### Analysis of bacterial community structure at the phylum level

**Figure 2:**
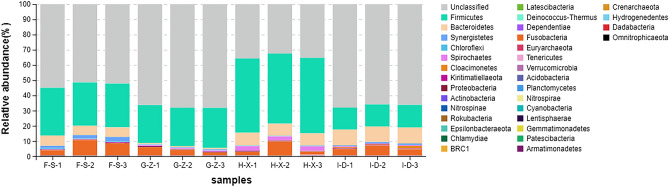
Stack diagram showing the species distribution of bacteria in pit mud from F-S, G-Z, H-X and I-D at the phylum level.

As shown in Fig. 2, at the phylum level, among phyla with a relative abundance of > 0.5%, there were 5 dominant bacterial phyla in the pit mud from F-S; these included Firmicutes (52.8%), Bacteroidetes (29.6%), Synergistetes (6.4%), Chloroflexi (2.1%) and Spirochaetes (0.6%). In the pit mud from G-Z, there were 3 dominant bacterial phyla, including Firmicutes (67.4%), Bacteroidetes (25.5%) and Synergistetes (0.9%). In the pit mud from H-X, there were 5 dominant bacterial phyla, including Bacteroidetes (48.2%), Firmicutes (34.4%), Synergistetes (8.0%), Chloroflexi (2.7%) and Cloacimonetes (2.6%). In the pit mud from I-D, there were 5 dominant bacterial phyla, including Firmicutes (66.7%), Bacteroidetes (14.7%), Synergistetes (10.3%), Kiritimatiellaeota (0.9%) and Chloroflexi (0.9%).

### Analysis of fungal community structure at the phylum level

**Figure 3:**
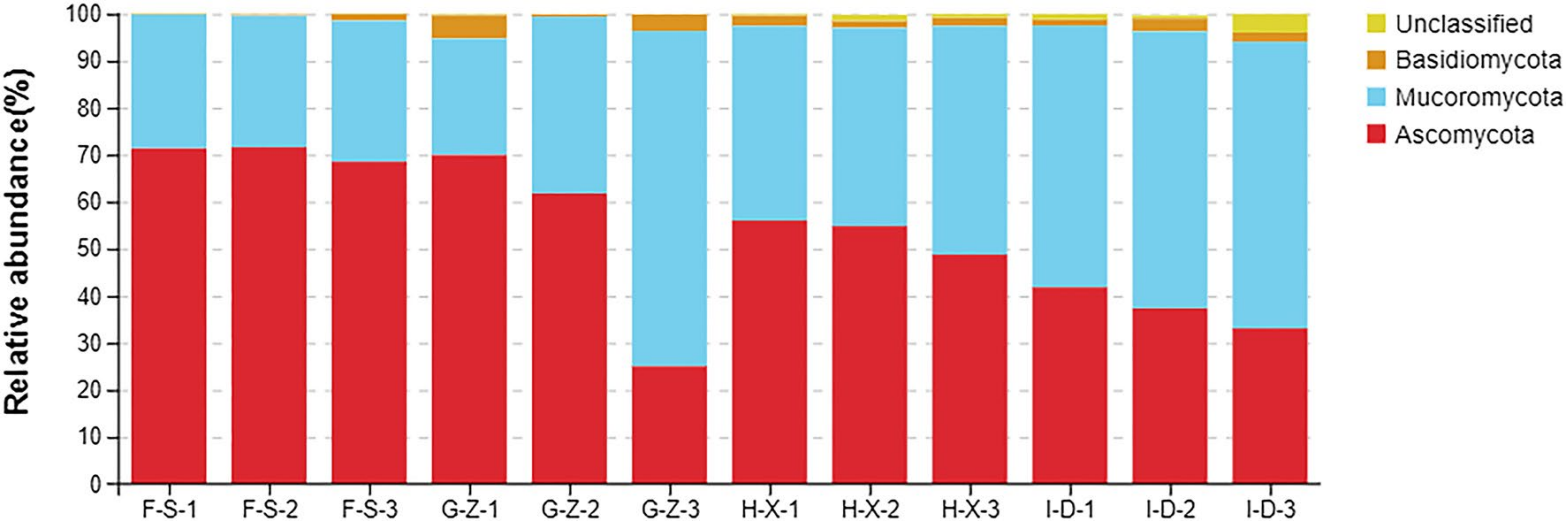
Stack diagram showing the species distribution of fungi in pit mud from F-S, G-Z, H-X and I-D at the phylum level.

As shown in Fig. 3, at the phylum level, among phyla with a relative abundance of > 0.5%, unclassified fungi in the pit mud from F-S accounted for 0.06% of the species present, and there were 3 dominant fungal phyla, including Ascomycota (70.5%), Mucoromycota (28.9%) and Basidiomycota (0.6%). In the pit mud from G-Z, unclassified fungi (unclassified) accounted for 0.2% of the species present, and there were 3 dominant fungal phyla, including Ascomycota (52.2%), Mucoromycota (44.6%) and Basidiomycota (3.0%). The unclassified fungi in the pit mud from H-X accounted for 0.9% of the species present, and there were 3 dominant fungal phyla, including Ascomycota (53.2%), Mucoromycota (44.1%) and Basidiomycota (1.8%). In the pit mud from I-D, unclassified fungi accounted for 2.0% of the species present, and there were 3 dominant fungal phyla, including Mucoromycota (58.5%), Ascomycota (37.4%) and Basidiomycota (2.0%).

### Analysis of bacterial community structure at the genus level

**Figure 4:**
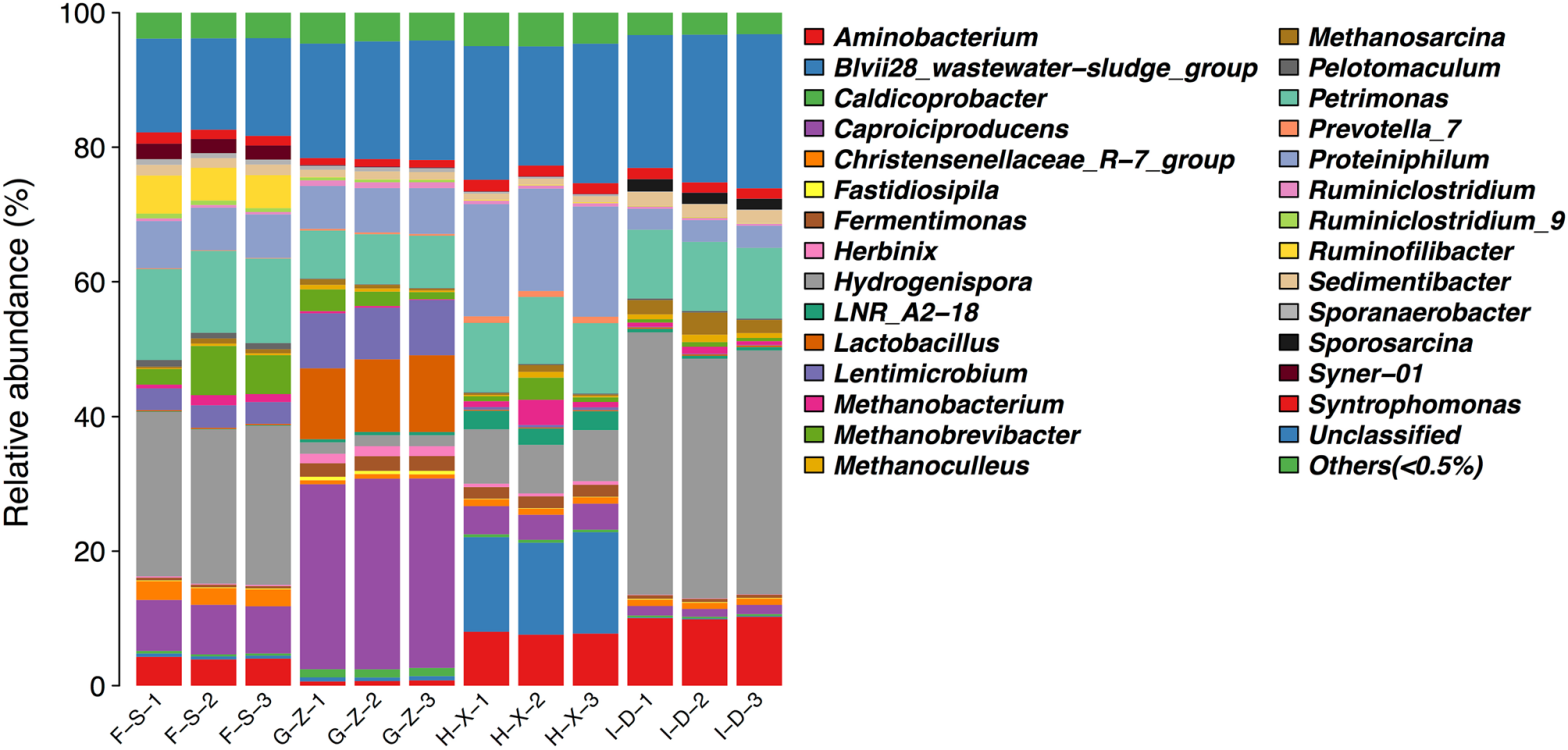
Stack diagram showing the species distribution of bacteria in pit mud from F-S, G-Z, H-X and I-D at the genus level.

As seen from Fig. 4, at the genus level, among genera with a relative abundance of > 1.0%, unclassified bacteria account for 14.02% of the bacteria present in the pit mud from F-S, and there are 11 dominant bacterial genera, including *Hydrogenispora* (23.67%), *Petrimonas* (12.75%), *Caproiciproducens* (7.32%), *Proteiniphilum* (6.61%), *Ruminofilibacter* (5.15%), *Aminobacterium* (4.06%), *Lentimicrobium* (3.27%), *Christensenellaceae R-7 group* (2.60%), *Syner-01* (2.16%), *Sedimentibacter* (1.53%) and *Syntrophomonas* (1.49%). In the pit mud from G-Z, unclassified bacteria account for 17.43%, and there are 11 dominant bacterial genera, including *Caproiciproducens* (28.00%), *Lactobacillus* (10.87%), *Lentimicrobium* (8.02%), *Petrimonas* (7.46%), *Proteiniphilum* (6.58%), *Fermentimonas* (2.13%), *Hydrogenispora* (1.64%), *Herbinix* (1.46%), *Caldicoprobacter* (1.22%), *Sedimentibacter* (1.12%), and *Syntrophomonas* (1.07%). In the pit mud from H-X, unclassified bacteria account for 19.44%, and there are 9 dominant bacterial genera, including *Proteiniphilum* (16.10%), *Blvii28_wastewater-sludge group* (14.27%), *Petrimonas* (10.21%), *Aminobacterium* (7.78%), *Hydrogenispora* (7.63%), *Caproiciproducens* (3.92%), *LNR_A2-18* (2.67%), *Fermentimonas* (1.76%) and *Syntrophomonas* (1.62%). In the pit mud from I-D, unclassified bacteria (*Unclassified*) accounted for 21.54%, and there were 8 dominant bacterial genera, including *Hydrogenispora* (36.92%), *Petrimonas* (10.33%), *Aminobacterium* (10.04%), *Proteiniphilum* (3.22%), *Sedimentibacter* (1.98%), *Sporosarcina* (1.68%), *Syntrophomonas* (1.56%) and *Caproiciproducens* (1.31%).

### Analysis of fungal community structure at the genus level

**Figure 5:**
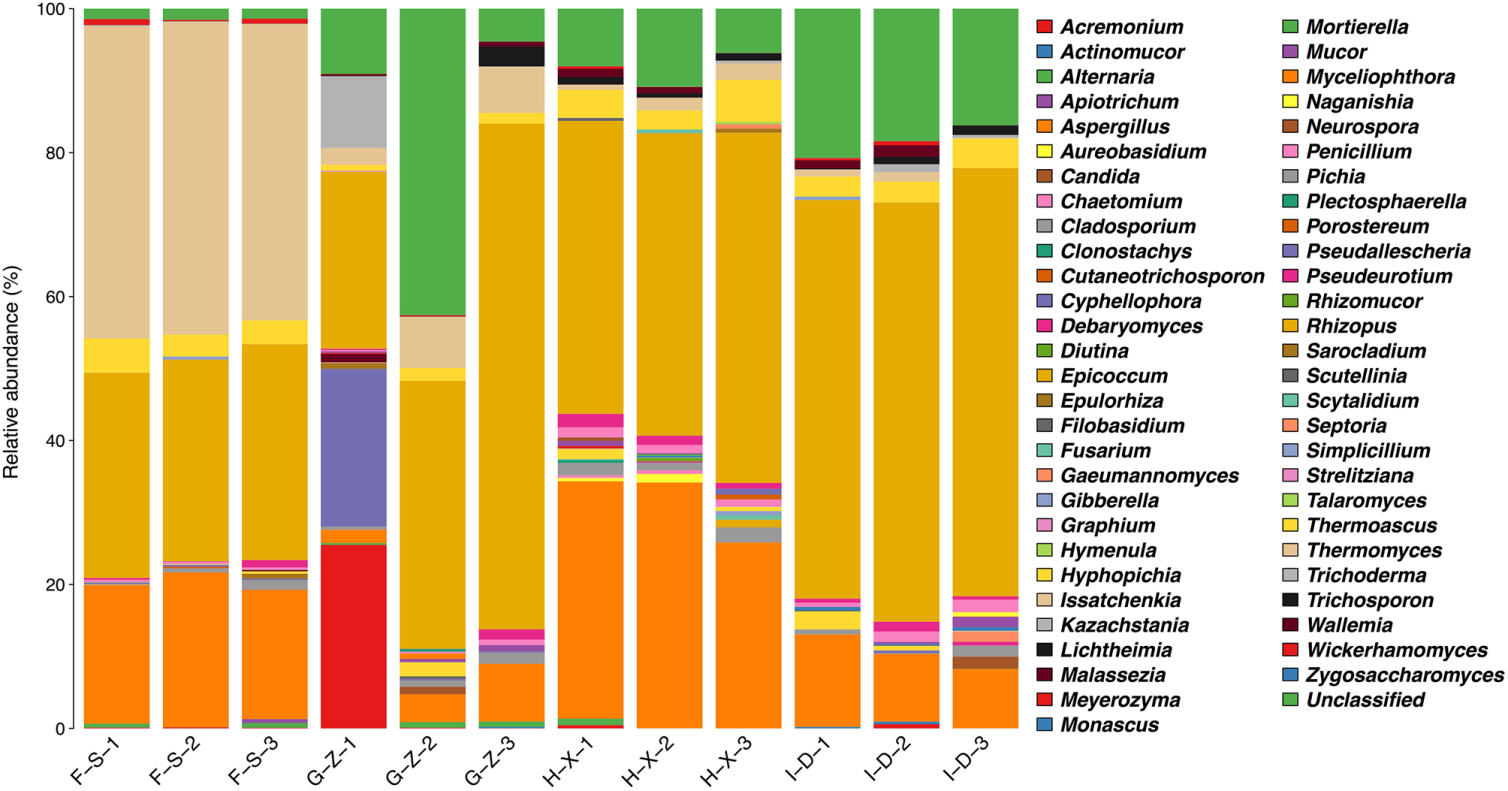
Stack diagram showing the species distribution of fungi in pit mud from F-S, G-Z, H-X and I-D at the genus level.

As seen from Fig. 5, at the genus level, among genera with a relative abundance of > 1.0%, unclassified fungi account for 1.5% of the fungi in the pit mud from F-S, and there are 4 dominant fungal genera, including *Thermomyces* (42.7%), *Rhizopus* (28.8%), *Aspergillus* (19.6%) and *Thermoascus* (3.7%). In the pit mud from G-Z, unclassified fungi account for 18.7%, and there are 7 dominant fungal genera, including *Rhizopus* (44.0%), *Acremonium* (8.5%), *Cyphellophora* (7.4%), *Thermomyces* (5.3%), *Aspergillus* (4.6%), *Trichosporon* (3.3%) and *Thermoascus* (1.4%). In the pit mud from H-X, unclassified fungi account for 8.3%, and there are 7 dominant fungal genera, including *Rhizopus* (43.8%), *Aspergillus* (31.0%), *Thermoascus* (4.1%), *Cladosporium* (1.6%), *Thermomyces* (1.6%), *Pseudeurotium* (1.3%) and *Penicillium* (1.2%). In the pit mud from I-D, unclassified fungi (*Unclassified*) account for 18.5%, and there are 5 dominant fungal genera, including *Rhizopus* (57.7%), *Aspergillus* (10.2%), *Thermoascus* (3.3%), *Penicillium* (1.2%) and *Hyphopichia* (1.0%).

### Composition and content of volatile compounds in pit mud

The composition and content of volatile compounds in pit mud are shown in Table 2.

**Table 2:** Composition and content of volatile compounds in pit mud.

Compound type	No	Compound name	Retention index	Retention time/min				Relative Content/%			
				Pit mud from F-S	Pit mud from G-Z	Pit mud from H-X	Pit mud from I-D	Pit mud from F-S	Pit mud from G-Z	Pit mud from H-X	Pit mud from I-D
Esters	1	Ethyl caproate	984	–	7.93	7.29	7.80	–	3.94	0.06	9.23
	2	Ethyl heptanoate	1083	–	10.819	10.598	9.866	–	0.14	0.06	0.65
	3	Ethyl 2-hydroxypropionate	848	8.509	11.099	10.896	10.221	5.43	5.65	1.05	0.35
	4	Butyl caproate	1183	11.137	12.575	12.461	12.054	0.55	0.12	0.05	0.7
	5	Hexyl butyrate	1183	11.25	–	–	12.122	0.17	–	–	0.21
	6	Ethyl caprylate	1183	11.863	13.023	12.929	12.586	1.47	0.64	0.16	3.07
	7	Isoamyl caproate	1218	12.562	–	–	13.095	0.1	–	–	0.63
	8	Butyl lactate	1047	14.137	–	–	–	0.46	–	–	–
	9	Amyl hexanoate	1282	–	–	–	14.192	–	–	–	0.66
	10	Propyl caprylate	1282	–	–	–	14.345	–	–	–	0.06
	11	Butyl 2-hydroxypropanoate	848	–	14.65	–	–	–	0.17	–	–
	12	Ethyl nonanoate	1282	–	–	–	14.665	–	–	–	0.12
	13	2-Ethylbutyric acid, 4-heptyl ester	1353	–	–	–	15.581	–	–	–	0.05
	14	Hexyl hexanoate	1381	15.856	16.095	16.073	15.987	0.3	0.27	0.27	10.68
	15	Heptyl acetate	1381	–	–	–	16.1	–	–	–	1.04
	16	Ethyl caprate	1381	16.362	16.537	16.522	16.444	0.14	0.33	0.1	0.44
	17	Diethyl succinate	1151	17.063	17.182	17.169	17.114	0.34	0.33	0.08	0.02
	18	Ethyl methoxyacetate	1159	17.734	17.816	17.809	17.764	0.28	0.24	0.04	0.07
	19	Ethyl phenylacetate	1259	18.739	18.791	18.782	18.755	0.67	0.46	0.3	0.26
	20	N-hexyl caprylate	1580	–	–	–	19.049	–	–	–	1.75
	21	Furfuryl acetate	1406	–	–	–	19.86	–	–	–	0.05
	22	Ethyl 3-Phenylpropionate	1359	20.122	20.136	20.134	20.122	0.75	0.33	0.29	0.3
	23	Caproic acid 4-octyl ester	984	–	–	20.209	–	–	–	0.04	–
	24	Allyl 2-ethylbutyrate	1019	–	–	20.331	20.321	–	–	0.2	0.08
	25	Ethyl methyl-4-pentenoate	920	20.332	20.22	–	–	0.27	0.04	–	–
	26	Caproic acid 2,2-Dimethylhexanoic acid	1197	–	20.338	–	–	–	0.1	–	–
	27	Octyl pelargonate	1878	–	–	–	20.383	–	–	–	0.1
	28	Octyl octanoate	1779	–	–	–	21.665	–	–	–	0.07
	29	Ethyl tetradecanoate	1779	22.134	22.136	22.134	21.537	0.16	0.18	0.14	0.16
	30	Ethyl pentadecanoate	1878	24.365	22.784	22.785	22.791	0.24	0.09	0.1	0.04
	31	Ethyl 13-methyl-tetradecanoate	–	–	22.968	–	–	–	0.13	–	–
	32	Ethyl tridecanoate	–	–	–	22.969	–	–	–	0.11	–
	33	Ethyl hexadecanoate	1978	24.549	24.549	24.548	22.974	0.91	0.77	0.49	0.78
	34	9-Hexadecenoicacid, ethylester	1986	–	–	–	24.86	–	–	–	0.04
	35	Ethyl 9-octadecenoic acid	2185	26.989	26.986	–	26.995	0.26	0.1	–	0.13
	36	Ethyl 9, 12-octadecadienoic acid	2193	27.486	27.486	–	–	0.34	0.21	–	–
	37	Ethylene acetate	2489	28.027	–	–	–	0.27	–	–	–
	38	Dibutyl phthalate	2037	29.358	–	–	–	0.23	–	–	–
Alcohols	1	1-Butanol	662	–	4.079	–	–	–	0.63	–	–
	2	3-Methyl-1-butanol	697	–	6.945	–	–	–	0.37	–	–
	3	1-Hexanol	2.56	9.173	11.314	11.142	10.505	2.56	1.83	0.41	0.95
	4	1-Octanol	1059	14.929	15.253	–	15.058	0.31	0.15	–	0.18
	5	6-Hendecanol	1277	17.355	–	–	–	0.09	–	–	–
	6	1-Decyl alcohol	1258	–	–	–	18.348	–	–	–	0.04
	7	Benzyl alcohol	1036	20.044	–	–	–	0.15	–	–	–
	8	Phenethyl alcohol	1136	20.506	20.514	20.513	20.489	1.31	1.75	0.64	0.18
	9	Triethylene glycol	1255	25.508	–	–	–	0.09	–	–	–
Acids	1	Acetic acid	576	12.69	13.387	13.312	13.033	3.76	3.65	2.15	1.1
	2	Propionic acid	676	–	14.95	14.903	14.759	–	0.06	0.58	0.07
	3	2-Methylpropionic acid	711	15.122	15.414	15.381	15.25	1.45	0.69	0.54	0.06
	4	Butyric acid	775	16.186	16.37	16.341	16.28	16.83	12.11	12.53	1.77
	5	2-Methylbutyric acid	775	–	–	16.45	–	–	–	0.01	-
	6	3-Methylbutyric acid	811	16.43	17.012	16.994	16.936	0.06	1.9	2.45	0.28
	7	3-Methylpentanoic acid	811	16.887	–	–	–	1.94	–	–	–
	8	Valeric acid	875	17.966	18.025	18.009	17.983	2.78	3.06	11.28	0.93
	9	α-Methyl phenylpropionic acid	–	18.805	–	–	–	0.05	–	–	–
	10	4-Methylpentanoic acid	910	18.918	18.945	18.939	–	0.14	0.18	0.19	–
	11	2-Methylpentanoic acid	910	–	–	18.414	–	–	–	0.04	–
	12	Hexanoic acid	974	19.52	19.538	19.544	19.524	43.82	50.43	50.63	39.99
	13	Heptanoic acid	1073	20.932	20.93	20.927	20.926	1.04	1.52	4.25	4.72
	14	Octanoic acid	1173	22.27	22.269	22.268	22.276	1.86	2.22	3.57	14.36
	15	Nonanoic acid	1272	23.542	23.538	23.539	23.541	0.1	0.07	0.15	0.44
	16	2-Phenethyl hexanoic acid	1657	–	–	–	23.66	–	–	–	0.19
	17	Capric acid	1372	24.755	24.751	24.751	24.755	0.09	0.15	0.18	0.79
	18	Benzoic acid	1150	26.495	26.597	26.596	–	0.33	0.11	0.11	–
	19	Phenylacetic acid	–	–	27.892	27.891	–	–	0.07	0.08	–
	20	Cis10-heptadecenoic acid	2075	–	–	–	27.789	–	–	–	0.14
	21	Tetradeconic acid	1769	29.209	–	–	–	0.12	–	–	–
	22	Palmitic acid	1968	32.198	32.198	32.197	–	2.36	0.14	0.11	–
Others	1	D-limonene	1018	–	6.055	–	–	–	0.16	–	–
	2	Tetramethylpyrazine	1121	–	13.758	13.681	–	–	0.38	0.14	–
	3	Cresol	1203	–	21.118	–	–	–	0.05	–	–
	4	Phenol	901	21.724	21.723	21.72	21.721	0.19	0.32	0.3	0.32
	5	P-methylphenol	1014	22.666	22.664	22.659	22.663	1.49	2.17	4.77	0.82
	6	3-Methylphenol	1014	–	–	–	22.755	–	–	–	0.03
	7	2,4-Di-tert-butylphenol	1555	–	–	–	25.224	–	–	–	0.16

A total of 77 volatile compounds were detected in the pit mud used for manufacturing Taorong-type Baijiu; these compounds included 38 esters, 9 alcohols, 22 acids and 7 other volatile compounds. Esters and acids are two of the dominant components of pit mud. There are significant differences in the types of esters and acids present, as well as in their content, in different layers of pit mud, while the differences in alcohols are not significant. The total content of volatile compounds in pit mud shows an upward-downward-upward trend with pit depth. 44 types of volatile compounds were found in the pit mud from layers F-S, 45 types were found in mud from layers G-Z, 39 types were found in mud from layers H-X, and 49 types were found in mud from layers I-D. There are also differences in the relative content of various components in the pit mud from layers F-S, G-Z, H-X and I-D. Ester compounds are the most volatile compounds present, and they present the highest content and variety in pit mud; 38 of these esters are the main contributors to the aroma of Taorong-type Baijiu, and there are 20, 20, 17 and 28 types of esters in the pit mud from layers F-S, G-Z, H-X and I-D, respectively. Ethyl caproate ranks first in relative content, with its content in the pit mud from layers G-Z, H-X and I-D being 3.94%, 0.06% and 9.23%, respectively. It is mainly generated under the synergistic action of various bacteria and enzymes^11^. Ethanol and acetic acid combine to form butyric acid, followed by the synthesis of caproic acid through the action of esterase. Subsequently, caproic acid is synthesized from ethanol^27^. Ethyl caproate is considered the key component contributing to the flavor and quality of Baijiu^19^. Ethyl caprylate and ethyl heptanoate rank second and third, respectively, with respect to the ester content of pit mud. Ethyl esters are present at the highest concentrations in pit mud; there are 13, 15, 12 and 14 different ethyl esters in the pit mud from F-S, G-Z, H-X and I-D, respectively, and these are the main esters in Taorong-type Baijiu. There was no significant difference in the ethanol compounds present in pit mud from different layers; 9 types of ethanol compounds were detected, including 6, 5, 2 and 4 types in the pit mud from layers F-S, G-Z, H-X and I-D, respectively. 1-Hexanol is the ethanol compound with the highest content in pit mud; its relative content in pit mud from layers F-S, G-Z, H-X and I-D is 2.56%, 1.83%, 0.41% and 0.95%, respectively. There are abundant acid compounds present in pit mud, and there are significant differences in the distribution of these compounds among the layers of pit mud. A total of 22 types of acid compounds were detected; 16, 25, 17 and 13 types were detected in the pit mud from layers F-S, G-Z, H-X and I-D, respectively. Among them, caproic acid, butyric acid, acetic acid, valeric acid, Octanoic acid and heptanoic acid are present at the highest levels. The content of caproic acid is extremely high; its relative content in pit mud from layers F-S, G-Z, H-X and I-D is 43.82%, 50.43%, 50.63% and 39.99%, respectively. Under the coupling action of Caproiciproducens and methanogens, acetic acid is produced by ethanol oxidation, and ethanol then reacts with butyric acid to produce caproic acid^28^. Caproic acid and ethyl caproate produced by pit mud fermentation are the main aromatic components of Taorong-type Baijiu^11^. A large amount of caproic acid is produced during fermentation; it then reacts with ethanol to produce ethyl caproate, the main aromatic component of Baijiu^29^. Acetic acid, butyric acid, heptanoic acid and Octanoic acid are present at the second highest levels, and they are the main organic acid components of Taorong-type Baijiu.

### Correlation between microbes and main volatile compounds in pit mud

**Figure 6:**
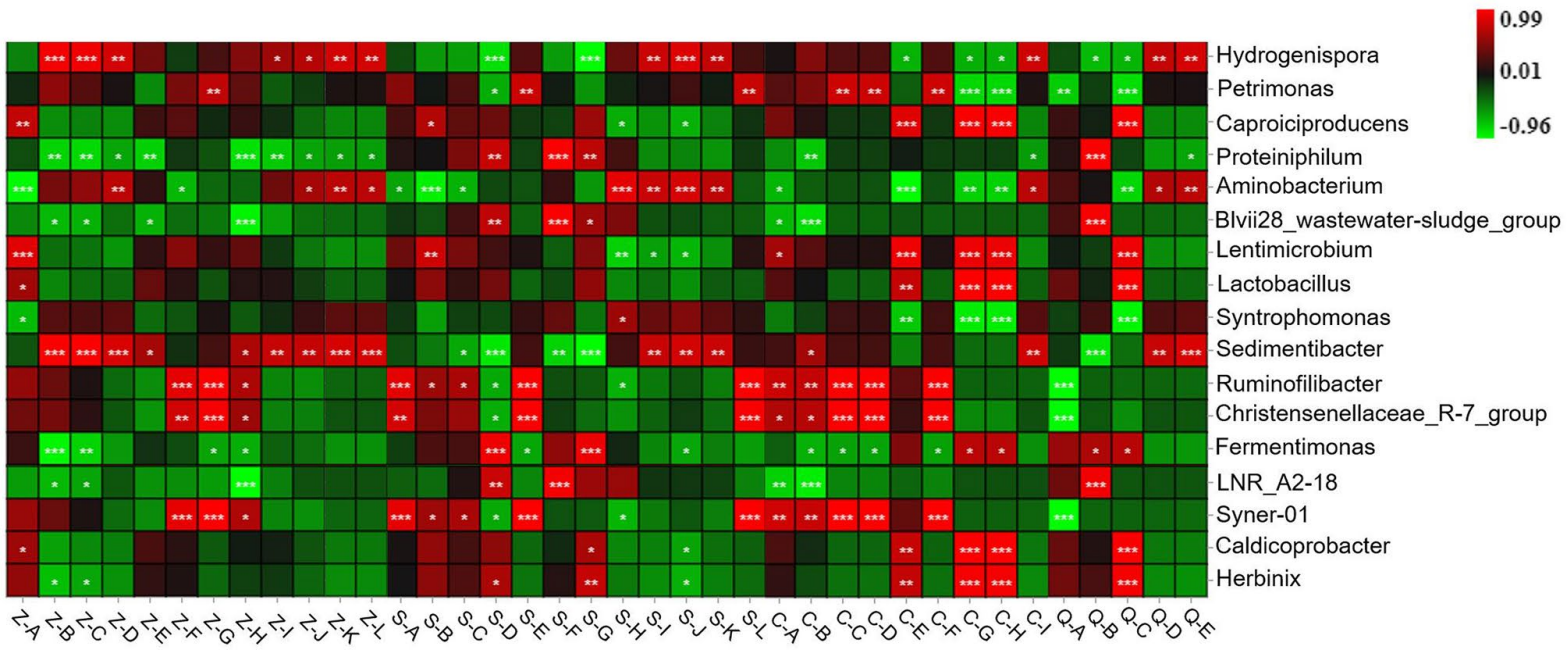
Heatmap showing the correlation between bacteria and the main volatile compounds in pit mud.

**Figure 7:**
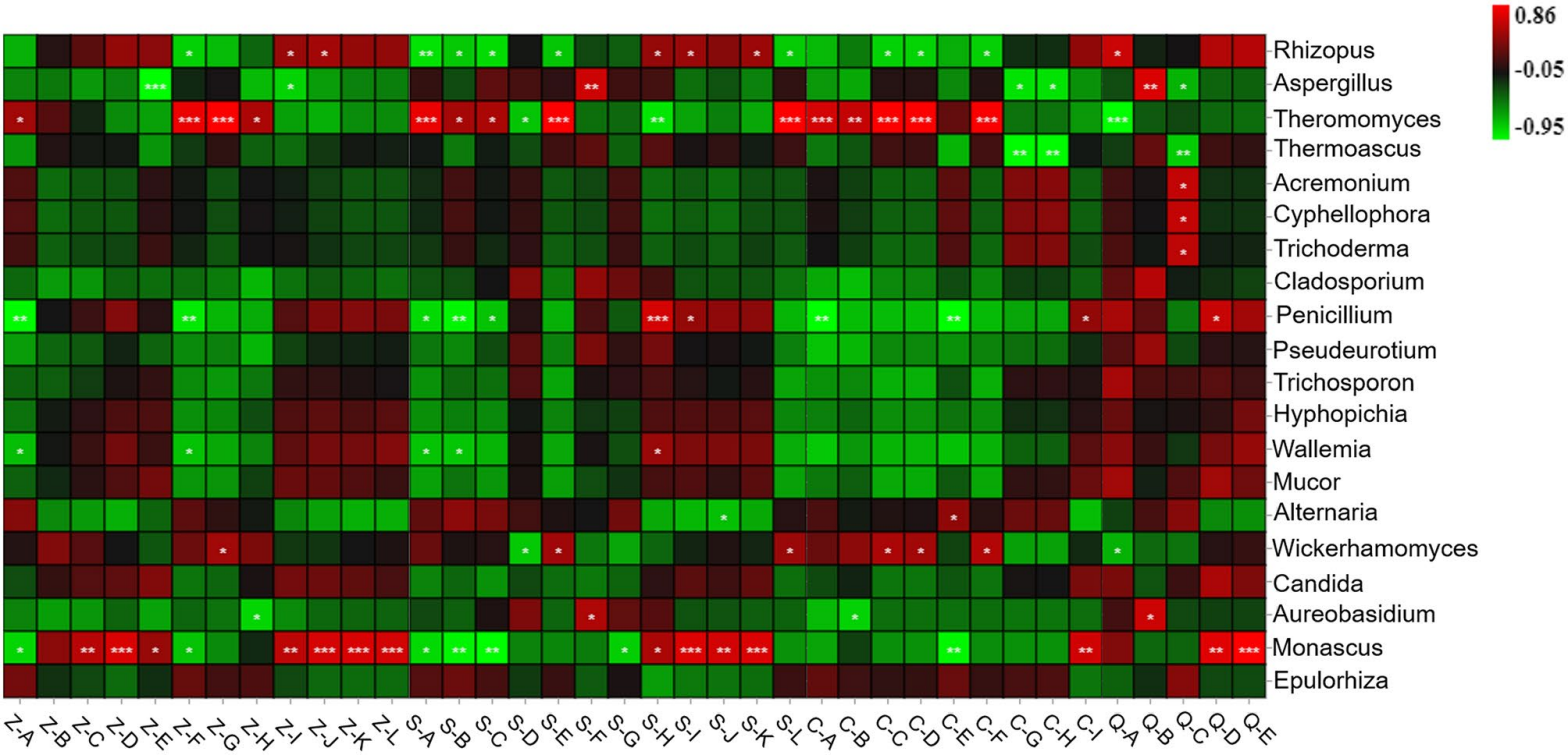
Heatmap showing the correlation between fungi and the main volatile compounds in pit mud. *Note*: Z-A, ethyl 2-hydroxypropionate; Z-B, butyl caproate; Z-C, ethyl caprylate; Z-D, hexyl hexanoate; Z-E, ethyl caprate; Z-F, ethyl phenylacetate; Z-G, ethyl 3-phenylpropionate; Z-H, ethyl hexadecanoate; Z-I, ethyl caproate; Z-J, ethyl heptanoate; Z-K, heptyl acetate; Z-L, N-hexyl caprylate; C-A, 1-hexanol; C-B, 1-octanol; C-C, 6-hendecanol; C-D, benzyl alcohol; C-E, phenethyl alcohol; C-F, triethylene glycol; C-G, 1-butanol C-H, 3-methyl-1-butanol C-I, 1-decyl alcohol; S-A, 2-methylpropionic acid; S-B, acetic acid; S-C, butyric acid; S-D, 3-methylbutyric acid; S-E, 3-methylpentanoic acid; S-F, valeric acid; S-G, caproic acid; S-H, heptanoic acid; S-I, Octanoic acid; S-J, nonanoic acid; S-K, capric acid; S-L, palmitic acid; Q-A, phenol; Q-B, p-methylphenol; Q-C, cresol; Q-D: 3-methylphenol; Q-E, 2,4-di-tert-butylphenol.

An analysis of the correlation between the main volatile compounds and the specific bacteria and fungi present in pit mud was conducted, and a correlation heatmap was obtained. As shown in Fig. 6, the bacterial genera that are closely correlated with the main volatile compounds present in pit mud include *Hydrogenispora*, *Aminobacterium*, *Lentimicrobium*, *Sedimentibacter*, *Ruminococcus*, *Christensenellaceae_R-7_group* and *Syner-01*. As shown in Fig. 7, the fungal genera that are closely correlated with the main volatile compounds present in pit mud include *Rhizopus*, *Thermomyces*, *Monascus* and *Penicillium*. Ethyl caproate is the main aromatic substance in Baijiu, and it has the highest correlation with *Sedimentibacter* and *Monascus*, followed by *Hydrogenispora* and *Rhizopus*. Sedimentibacter can synthesize caproic acid, butyric acid, acetic acid, hexanol, ethanol and butanol using carbon sources and protein as substrates and can generate ethyl caproate. Acetic acid was positively correlated with the presence of *Syner-01*, *Ruminococcus*, *Lentimicrobium*, *Caproiciproducens* and *Thermomyces* and negatively correlated with the presence of *Aminobacterium*, *Monascus* and *Penicillium*. Hexanol, octanol, 6-hendecanol and benzyl alcohol were positively correlated with the presence of *Syner-01*, *Christensenellaceae_R-7_group*, *Ruminococcus* and *Thermomyces.* This shows that the microbial community structure in pit mud has a certain influence on the flavor and quality of Baijiu.

## Conclusion and discussion

In this study, HTS technology was used to analyze the bacterial community structure of pit mud used for manufacturing Taorong-type Baijiu at Henan Yangshao Liquor Co., LTD. The volatile compounds in the pit mud used for manufacturing Taorong-type Baijiu were analyzed qualitatively using a method that combined HS-SPME and GC–MS in an attempt to understand the composition of volatile compounds in pit mud.

Five, 3, 5 and 5 dominant bacterial phyla were found in the pit mud from layers F-S, G-Z, H-X and I-D, respectively. The common dominant bacterial phyla shared in all layers included Firmicutes, Bacteroidetes and Synergistetes. Firmicutes was the dominant bacterial phylum in the pit mud from layers F-S, G-Z and I-D, and Bacteroidetes was the dominant bacterial phylum in the pit mud from layer H-X. At the phylum level, there was no significant difference in the bacterial community structure of pit mud at different spatial positions. With an increase in depth within the pits, the content of Firmicutes shows an upward-downward-upward trend, reaching 66.7% in layer I-D. Firmicutes possess a cell wall, and most of them can produce endophytic spores and resist extreme environments. In addition, the flora of Firmicutes have the function of producing caproic acid and reducing lactic acid^30^. Synergistetes content shows a decreasing-increasing trend with depth, with the highest content of 10.3% in layers I-D. These bacteria can degrade amino acids, and some of them are specific anaerobes. Deng et al.^31^ adopted HTS technology to explore the microbial community structure in 5-year-old and 30-year-old pit mud from a wine company in Sichuan and found that Firmicutes, Bacteroidetes, Synergistetes, Spirochaetes and Chloroflexi were the dominant bacterial genera. In our study, there were 3 dominant fungal phyla, including Ascomycota, Mucoromycota and Basidiomycota, in the pit mud from layers F-S, G-Z, H-X and I-D. It can be seen that, at the phylum level, the same structural composition of fungal phyla is found in different layers of pit mud. The contents of Ascomycota and Mucoromycota were highest, and they were absolutely dominant fungal phyla in each layer of pit mud. Overall, at increased depth within pit mud, the relative abundance of Ascomycota shows a downward trend, while that of Mucoromycota shows an upward trend.

There were 11, 11, 9 and 8 dominant bacterial genera in the pit mud from layers F-S, G-Z, H-X and I-D, respectively. The dominant microbial genera shared among these layers include *Hydrogenispora*, *Petrimonas*, *Caproiciproducens*, *Proteiniphilum* and *Syntrophomonas*. *Hydrogenispora* is the dominant microbial genus in the pit mud from F-S and I-D, *Caproiciproducens* is the dominant microbial genus in the pit mud from G-Z, and *Proteiniphilum* is the dominant microbial genus in the pit mud from H-X. *Syner-01*, *Pelotomaculum*, *Ruminofilibacter* and *Ruminiclostridium 9* are the uniquely dominant microbial genera in the pit mud from F-S; *Lactobacillus*, *Herbinix*, *Ruminiclostridium*, *Caldicoprobacter* and *Fastidiosipila* are the uniquely dominant microbial genera in the pit mud from G-Z; LNR_A2-18 and *Prevotella_7* are the uniquely dominant microbial genera in the pit mud from H-X, and *Sporosarcina* is the uniquely dominant microbial genus in the pit mud from I-D.

At the genus level, there is heterogeneity in the microbial community structure of pit mud at different spatial levels. *Hydrogenispora*, *Caproiciproducens*, *Sedimentibacter* and *Syntrophomonas* belong to the family Clostridiaceae. Clostridia flora can synthesize caproic acid, butyric acid, acetic acid, hexanol, ethanol and butanol using carbon sources and protein as substrates, and they can also generate ethyl caproate^32^. In the pits, Clostridia and Bacteroidia are present in high proportions (51.1% and 29.5%, respectively), and the content of Clostridia is highest in the pit mud from I-D, reaching 63.86%, indicating that the tested pit mud samples are in the mature state. Clostridia can use ethanol and acetic acid to generate acetoacetic acid^33^, and it is one of the key microbial groups that promotes the synthesis of short- and medium-chain fatty acids such as butyric acid and caproic acid^34^. These microbes can be easily screened from pit mud. *Hydrogenispora*, *Sedimentibacter*, *Petrimonas*, *Syntrophomonas* and *Aminobacterium* have a high content in the pit mud from layer I-D. *Hydrogenispora* can produce acetate, ethanol and H_2_^35^. *Sedimentibacter* and *Aminobacterium* can ferment amino acids^32^ to generate ammonium nitrogen through metabolism, and this could provide nitrogen sources for the growth of other microbes^36^. In addition, they have the function of degrading lactic acid; most members of Petrimonas can use glucose to produce H_2_ and CO_2_ or acetic acid and propionic acid. *Aminobacterium*, *Syntrophomonas* and *Petrimonas* play a positive role in the maturation of pit mud^37^. *Caproiciproducens* is a significant flora in pit mud. Because the caproic acid produced by *Caproiciproducens* inhibits the growth of lactic acid bacteria and the quality of pit mud is closely correlated with pH, reduction in the growth of lactic acid bacteria can improve the quality of pit mud. *Lactobacillus* is the unique dominant bacterial genus in the pit mud from layers G-Z, and its metabolism can produce aromatic substances of Nongxiang-type (strong aroma) Baijiu. However, the *Lactobacillus* content of the pit mud from G-Z was high, reaching 10.87%. Accumulation of lactic acid will increase the content of ethyl lactate in Baijiu and reduce its quality^29^. In addition, the content of *Lactobacillus* in pit mud decreases significantly as the pit mud matures^38^. Hu et al.^39^ also found that the *Lactobacillus* content of high-quality pit mud decreased significantly and that the content of core bacteria such as *Aminobacterium* increased significantly. Therefore, a moderate amount of lactic acid bacteria should be present in pit mud^36^.

There were 4, 7, 7 and 5 dominant fungal genera in the pit mud from layers F-S, G-Z, H-X and I-D, respectively. *Thermomyces* is the dominant fungal genus in pit mud from F-S, and its content decreases with increasing pit mud depth. *Rhizopus* is the dominant fungal genus in the pit mud from G-Z, H-X and I-D, and its content shows an upward trend with increasing pit mud depth. *Thermomyces* is the dominant mold in the pit mud used for manufacturing Daqu jiu and in the brewing environment. It has favorable comprehensive enzyme activity characteristics and can be used as an important microbial index for evaluation of the brewing environment, monitoring, determination of the appropriate storage period and quality evaluation of Daqu products^40^. In addition, it has strong thermal stability and can maintain stable catalytic efficiency under the high-temperature conditions used in the fermentation process of Baijiu^41^. *Rhizopus* is the main contributor to the proteins analyzed in metabonomics. It plays a saccharification role by secreting three glycosidases and two glycosyltransferases^42^. It is the crucial flora for the saccharification of distiller's yeast and contributes to improving the saccharification agent for food fermentation. *Aspergillus* has been extensively applied in the brewing industry. It has a certain level of acid resistance, a strong ability to produce amylase and protease and to metabolize organic acids, and can secrete glucoamylase^43^. Therefore, it plays an important role in production and aroma generation of Baijiu.

A total of 77 volatile compounds were detected in the pit mud samples analyzed in this study; 44, 45, 39 and 49 types of volatile compounds were found in pit mud from layers F-S, G-Z, H-X and I-D, respectively. These volatile compounds mainly include esters, acids and alcohols. Esters and acids are the two main components of these pit mud samples. There are significant differences in the esters and acids present in different layers of pit mud, as well as in their relative content, while the differences in alcohols are not significant. Ethyl caproate, 1-hexanol and caproic acid are the esters, alcohols and acids, respectively, that are present in the highest amounts in these pit mud samples. A correlation analysis between the microbes and the volatile compounds present in the pit mud samples was also conducted, and the correlation heatmap clearly showed that there is a correlation between specific microbes and the presence of specific compounds. Moreover, *Lentimicrobium*, *Syner-01* and *Blvii28_wastewater-sludge groups* were found in the pit mud used for manufacturing Taorong-type Baijiu for the first time. This work provides a resource for establishing a microbial information database for Taorong-type Baijiu. The findings of this study also provide theoretical support for interventions designed to improve the quality of pit mud and enhance the flavor and quality of Taorong-type Baijiu.

## Supplementary Information

Supplementary Information.
