# Supplementary material for: Analysis of microbial community structure and volatile compounds in pit mud used for manufacturing Taorong-type Baijiu based on high-throughput sequencing
Source: Sci Rep. 2022 May 5;12:7347. doi: 10.1038/s41598-022-10412-8 (PMC9072327; doi:10.1038/s41598-022-10412-8)
Supplement: Supplementary file 1 — Supplementary Information. [file 41598_2022_10412_MOESM1_ESM.docx]

**Analysis of Microbial Community Structure and Volatile Compounds in Pit Mud Used for Manufacturing Taorong-type Baijiu Based on High-throughput Sequencing**

**Yanbo Liu^1,2,3,4,5^, Mengxiao Sun^1,4,5^, Pei Hou^6^, Wenya Wang^1,4,5^,**

**Xiangkun Shen^7^, Lixin Zhang^3^, Suna Han^2^, Chunmei Pan^1,4,5🖂^**

1 College of Food and Biological Engineering(Liquor College), Henan University of Animal Husbandry and Economy,Zhengzhou , 450046, China, 2 Postdoctoral Programme, Henan Yangshao Distillery Co., Ltd., Mianchi 472400, China, 3 School of life sciences, Henan University,Kaifeng 475004, China,4 Henan Liquor Style Engineering Technology Research Center, Henan University of Animal Husbandry and Economy, Zhengzhou , 450046, China,5 Zhengzhou Key Laboratory of Liquor Brewing Microbial Technology, Henan University of Animal Husbandry and Economy, Zhengzhou , 450046, China ,6 School of Food and Bio-engineering, Zhengzhou University of Light Industry, Zhengzhou , 450000, China7.Henan Food Industry Science Research Institute Co., Ltd., Zhengzhou 450003, China)

Corresponding author **🖂** [sige518888@163.com](mailto:sige518888@163.com)

**supplementary information**

**Fig. 8 GC-MS diagram of volatile components in the upper layer of Taorong liquor cellar mud**

**Fig. 9 GC-MS diagram of volatile components in the middle layer of Taorong liquor cellar mud**

**Fig.10 GC-MS diagram of volatile components in the lower layer of Taorong liquor pit mud**

**Fig. 11 GC-MS map of volatile components in the bottom layer of Taorong liquor cellar mud**
